# CPAN: Validation of a novel transdiagnostic dimensional psychosis rating scale

**DOI:** 10.1192/j.eurpsy.2025.422

**Published:** 2025-08-26

**Authors:** L. Hermán, E. Vass, J. M. Réthelyi

**Affiliations:** 1 Department of Psychiatry and Psychotherapy, Semmelweis University, Budapest, Hungary

## Abstract

**Introduction:**

Psychiatric assessment of psychotic disorders has traditionally relied on categorical classification systems, but there is a shift towards a dimensional approach in DSM-5 and ICD-11. Schizophrenia is increasingly viewed as a spectrum disorder, with genetic studies indicating shared risk factors among schizophrenia, schizoaffective disorder, and bipolar disorder. However, there is currently no widely used transdiagnostic dimensional assessment tool in clinical practice. At Semmelweis University we have developed a scale which takes into account four major symptom groups (catatonia, affective-, positive and negative symptoms) and several important “specifiers” (disorganisation, bipolarity, prodromal symptoms, childhood onset, etc.). Clinicians should assess their patients with CPAN based on the long-term clinical presentation, contrary to PANSS and other cross-sectional tools, since our theory is that long-term traits represent underlying “biology” in a more precise manner than the rapidly changing status of patients, and therefore should show higher correlation with biomarkers like genetic and imaging data.

**Objectives:**

We aimed to test the clinical usability of CPAN and its alignment with DSM-5 diagnostic categories and medication correlations in outpatient settings. Additionally, we planned a validation process to assess the tool’s validity, interrater reliability, and test-retest reliability.

**Methods:**

In our pilot study, six clinicians assessed 104 outpatient patients using CPAN, analyzing DSM-5 diagnoses and medications. Patients were clustered into four groups based on leading symptoms. In the validation study, 100 inpatients with severe psychotic symptoms will be assessed three times by two raters—one from the clinical team and one independent. We will compare CPAN’s validity to PANSS results and assess test-retest reliability with three assessments.

**Results:**

The pilot study demonstrated that CPAN is user-friendly, taking 1-2 minutes for familiar clinicians to complete. Four symptom clusters were identified: 1) schizophrenia with catatonic symptoms, 2) schizophrenia without catatonic symptoms, 3) schizoaffective disorder with negative symptoms, and 4) schizoaffective disorder without negative symptoms/bipolar disorder. Prescription patterns were correlated with symptom groups, but detailed analysis was limited due to the small sample size. Validation results are pending.

**Conclusions:**

CPAN is a practical tool for assessing long-term symptom presentation in patients with psychotic disorders. Widespread use of this scale could provide valuable real-life data linking symptoms to medication use and clinical outcomes. The ongoing validation study will further establish the scale’s validity and reliability

**Disclosure of Interest:**

None Declared

